# The Role of Family Pathology in Noma: A Scoping Review of Household‐Level Risk Factors in Sub‐Saharan Africa

**DOI:** 10.1155/jotm/4767171

**Published:** 2026-05-27

**Authors:** Mujtaba Bala, Mohammed Adam Sheikh Abdullahi, Ramat Oyebunmi Braimah, Abdurrazaq Olanrewaju Taiwo, Ibrahim Kayode Suleiman, K. Hakki Karagozoglu

**Affiliations:** ^1^ Department of Dental and Maxillofacial Surgery, Usmanu Danfodiyo University Teaching Hospital, Sokoto, Nigeria, udusok.edu.ng; ^2^ Department of Oral and Maxillofacial Surgery, Faculty of Dental Sciences, College of Health Science, Usmanu Danfodiyo University, Sokoto, Nigeria, udusok.edu.ng; ^3^ Department of Oral and Maxillofacial Surgery, University of Maiduguri Teaching Hospital, Maiduguri, Borno State, Nigeria, unimaid.edu.ng; ^4^ Department of Oral and Maxillofacial Surgery/Oral Pathology, Amsterdam UMC, Vrije Universiteit Amsterdam, Amsterdam, the Netherlands, vu.nl

**Keywords:** cancrum oris, child health, family pathology, Noma, risk factors, scoping review

## Abstract

**Background:**

Noma is a neglected tropical disease that predominantly affects young children in sub‐Saharan Africa, characterized by rapid orofacial tissue destruction and high mortality. While malnutrition and infections are well‐recognized risk factors, less attention has been given to family‐level and psychosocial determinants.

**Objectives:**

To synthesize existing evidence on the role of family pathology in Noma and identify household‐level risk factors affecting disease development and outcomes.

**Methods:**

A scoping review was conducted in accordance with PRISMA‐ScR guidelines. PubMed and MEDLINE were searched through May 2024 for Noma‐specific literature and broader child health studies related to family environment. Data extraction focused on predefined family pathology themes. Narrative synthesis was performed due to the heterogeneity of study designs.

**Results:**

Thirty‐five studies met the eligibility criteria, including Noma‐specific case series, epidemiological reports, and child health literature. Eleven family pathology themes were identified: child developmental stage, family structure, living area, family income, family size, parental education, parental viability, marital conflict, family separation, primary caregiver, and caregiving quality. Poverty, large family size, limited caregiving quality, and psychosocial instability were interconnected, increasing the vulnerability to Noma.

**Conclusion:**

Family dysfunction and socioeconomic deprivation contribute significantly to Noma risk. Prevention strategies should integrate family‐level interventions, including caregiver education, birth spacing, economic support, and community health outreach. Addressing both biomedical and familial determinants is essential for reducing disease burden.

## 1. Introduction

Noma (cancrum oris) is a necrotizing orofacial infection that primarily affects children aged 2–6 years, which leads to rapid facial tissue destruction [1]. Commonly referred to as the “face of poverty” in sub‐Saharan Africa, it mainly affects economically disadvantaged and socially marginalized children [1, 2]. It is estimated that 30,000–40,000 new cases occur annually, with mortality during the acute stage exceeding 80% [3–5]. Those who survive often face severe facial deformities, functional impairments, and profound stigma [6].

While biomedical risk factors, such as severe malnutrition, infections (measles, malaria, HIV), poor oral hygiene, and immunosuppression, are well known, increasing attention is being directed toward the underlying familial and social determinants [1, 7]. Noma is therefore regarded not merely as a clinical condition but as a “disease of poverty” and neglect, closely associated with adverse living and caregiving environments [4–6].

Children who develop Noma often live in remote rural areas with limited resources and restricted access to healthcare services. They typically suffer from chronic undernutrition and untreated illnesses, closely related to their family circumstances [1, 7]. In large families, particularly those with high maternal parity, children born later may be at heightened risk due to resource dilution or reduced caregiver attention [4, 7]. Researchers are increasingly examining not only economic deprivation but also the role of family pathology and dysfunctional aspects of family life, such as chronic stress, disorganization, or failure to meet children’s basic needs, which can allow a minor oral ulcer to escalate into gangrene [1, 8–10]. These dynamics fall within the framework of systems theory, which defines family pathology as the breakdown of essential familial roles [8].

Family pathology can be classified into several types, each of which may affect child health and development differently [8]. Structural dysfunctions include parental absence, family separation, polygamous or extended households, and large sibship size, which may limit supervision and access to care. Psychosocial dysfunctions encompass marital conflict, domestic violence, parental depression, and chronic stress, all of which can impair caregiving quality and negatively impact child well‐being [9]. Functional or caregiving‐related dysfunctions involve inadequate nutrition, poor hygiene, inconsistent care, and overburdened caregivers. These dimensions often interact, amplifying risk factors for diseases such as Noma [8, 9].

The concept of family pathology encompasses failures in nurturance, enculturation, and guidance, key family functions as outlined by systems theory [8]. In the context of Noma, this may involve food insecurity, inattentive caregiving, or unstable family structures that increase the risk of malnutrition, oral infections, and delayed treatment [1, 4, 10].

These observations align with broader evidence that the family is a central setting for health promotion and that family behaviors and structures significantly influence child health outcomes [11–13]. When families fail to provide adequate nutrition, engage in preventive health practices, or offer a stable and nurturing environment, children face an increased risk of severe diseases of neglect [1, 4, 13].

Although few studies have examined family pathology in Noma, evidence from other facial deforming conditions, such as pediatric burns, traumatic facial injuries, and congenital craniofacial anomalies, demonstrates that family dynamics significantly influence disease outcomes and psychosocial adjustment. In cases of severe burns, inconsistent caregiving, parental stress, and poor family support have been associated with delayed recovery, increased complications, and emotional distress in children [14, 15]. Similarly, traumatic facial injuries often result in adverse psychological outcomes when family resources and support are limited [16]. These findings suggest that dysfunctional family environments may amplify the impact of Noma on children, emphasizing the importance of examining familial determinants as part of disease prevention and rehabilitation strategies.

Beyond the immediate health consequences, the family context plays a crucial role in how children cope with Noma and shapes long‐term outcomes for survivors. A scoping review by Onu et al. found that approximately one in three Noma survivors experiences mental health conditions and that social disadvantage and supernatural explanations for the disease are common within affected families [10]. Stigma may also affect family members, who risk being shunned by their communities, further underscoring that Noma is not merely a biomedical issue but deeply entangled with family and societal dynamics [1, 10].

The aim of this review is to explore how family pathology influences Noma in children, focusing on eleven key themes, including family structure, socioeconomic conditions, and caregiving patterns. By integrating findings from Noma‐specific studies and broader child health research, we aim to identify how family environments contribute to disease development and to highlight strategies for prevention and intervention.

## 2. Methods

### 2.1. Search Strategy

A scoping review was conducted in accordance with the Preferred Reporting Items for Systematic Reviews and Meta‐Analyses extension for Scoping Reviews (PRISMA‐ScR) guidelines. PubMed and MEDLINE were systematically searched from inception through May 2024. No restrictions on publication date were applied.

The core search strategy focused on identifying Noma‐specific literature using the terms “Noma” and “cancrum oris” in combination with family‐ and environment‐related concepts such as “family,” “socioeconomic,” “risk factors,” “parental,” “caregiver,” “poverty,” and “psychosocial.”

To ensure comprehensive coverage of family‐related determinants, additional searches were performed using broader child health terms (e.g., “family size malnutrition child,” “parental education child health”). These supporting searches were intended to capture studies addressing family environment and child development more generally, in order to fill conceptual gaps not well covered by Noma‐specific research.

Reference lists of included Noma articles were also hand‐searched to identify additional eligible studies. All searches were conducted through May 2024, and the complete search strings are provided in Supporting Information 1.

Given the limited research on family pathology in Noma, additional studies from broader child health literature were included. These studies addressed family environment, caregiving, nutrition, and psychosocial factors and were used to complement Noma‐specific research and provide context for interpreting familial determinants of disease.

### 2.2. Eligibility Criteria

#### 2.2.1. Studies Were Included if They Met All of the Following Criteria

1. Peer‐reviewed and indexed in PubMed.
2. Investigated risk factors for Noma, including familial, socioeconomic, and caregiving aspects.
3. Case–control studies, cohort studies, case series, clinical reports, or reviews.
4. Child health studies examining the role of family environment, nutrition, or development, used to complement Noma‐specific literature.

#### 2.2.2. Studies Were Excluded if They Were

1. Not peer‐reviewed or published in nonindexed journals.
2. Gray literature, media reports, or conference abstracts without full text.
3. Non‐English publications.

### 2.3. Study Selection

All studies were evaluated against the eligibility criteria described above. All titles and abstracts retrieved from the search were independently screened by two reviewers (M.B. and M.A.). Full texts of potentially relevant studies were then evaluated against the eligibility criteria. Discrepancies were resolved through discussion, with a third reviewer consulted when necessary.

In addition, the reference lists of the included Noma studies were screened to identify further eligible publications. This ensured that both Noma‐specific research and broader child health literature addressing family and caregiving themes were comprehensively captured.

**FIGURE 1:**
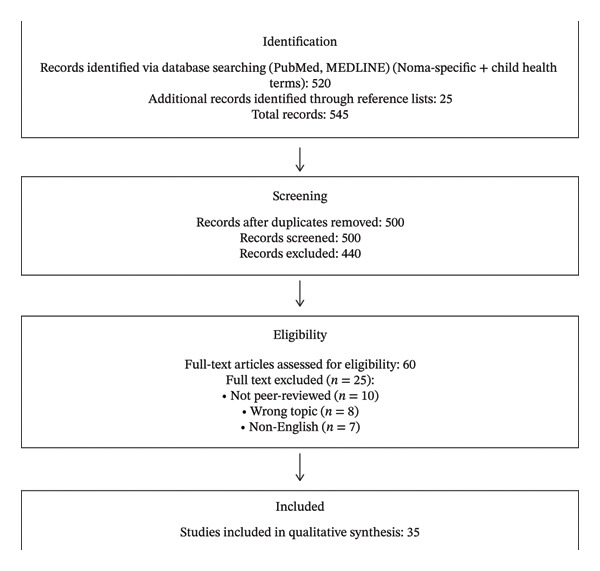
The PRISMA‐ScR flow diagram of the study selection process.

A PRISMA‐ScR flow diagram summarizing the selection process is presented in Figure 1.

### 2.4. Data Extraction and Management

Data were extracted into a standardized form capturing study design, setting, population characteristics, and key findings relevant to predefined family pathology themes. Noma‐specific studies were prioritized, while broader child health research was integrated to fill thematic gaps. Comparative studies (e.g., polygamous versus monogamous households) were emphasized where relevant. Data mapping was performed across eleven predefined themes, with cross‐referencing where thematic overlaps occurred.

### 2.5. Synthesis of Results

Due to the heterogeneity in study designs and limited quantitative data, a narrative synthesis was employed. Findings were triangulated between Noma‐specific studies and broader child health literature to strengthen the validity of interpretations. The results were structured around eleven thematic domains: child’s developmental stage, family structure, living area, income, family size, parental education and health, marital conflict, family separation, primary caregiver, and caregiving quality. Interrelationships between these themes, such as the compounding effects of poverty and family size, were also explored.

## 3. Results

### 3.1. Overview of Included Studies

A total of 35 studies met the eligibility criteria, encompassing both Noma‐specific research and broader child health literature. The included studies varied in design, including one prospective case–control study, several retrospective hospital‐based case series from Nigeria, and narrative reviews on the epidemiology and sociodemographic context of Noma. The majority of studies originated from West African countries, particularly Nigeria and Niger.

Consistently, these studies identified poverty and malnutrition as dominant risk factors for Noma. Some studies also provided additional data on family size, parental occupation, and educational attainment. For example, Oji observed that all Noma patients in his Nigerian cohort came from lower socioeconomic groups and attributed regional variations in Noma incidence to differences in familial feeding practices [17]. Similarly, Adeniyi and Awosan reported on 159 Noma cases in Northwestern Nigeria, with 87% of patients aged 1–5 years, highlighting early childhood as a critical window of vulnerability [18]. However, most hospital‐based studies primarily focused on biomedical risk factors and provided limited information on family structure or caregiving practices.

To address these gaps, evidence from broader child development and family psychology research was integrated. This included meta‐analyses on the effects of marital conflict on child health, longitudinal studies on parental depression, comparisons between children from polygamous and monogamous families, and analyses of socioeconomic influences on parental time investment. These broader studies offered critical context for interpreting the familial environments associated with Noma.

### 3.2. Family Pathology Themes

#### 3.2.1. Eleven Recurrent Themes Were Identified

##### 3.2.1.1. Child’s Developmental Stage

Noma primarily affects toddlers and preschoolers (∼3–4 years old), a critical period for cognitive and emotional development that requires intensive caregiving.

##### 3.2.1.2. Family Structure

Many cases originate from complex family systems, such as polygamous or extended families, where caregiving responsibilities may be dispersed.

##### 3.2.1.3. Living Area

Affected families predominantly reside in rural, isolated areas with poor sanitation and limited access to healthcare services.

##### 3.2.1.4. Family Income

Extreme poverty was universally reported and consistently identified as a key risk factor for Noma.

##### 3.2.1.5. Family Size

Large sibship size and high maternal parity were consistently associated with increased risk, likely due to resource dilution.

##### 3.2.1.6. Parental Education

Low or absent maternal education correlated with reduced health literacy and poor access to preventive healthcare.

##### 3.2.1.7. Parental Viability

Many children experienced parental absence due to death, illness, or labor migration.

##### 3.2.1.8. Marital Conflict

Domestic instability, often linked to polygamous rivalries or marital discord, was common and associated with negative child health outcomes.

##### 3.2.1.9. Family Separation

Frequent parental absence, whether due to labor migration or marital breakdown, was linked to diminished child supervision and care.

##### 3.2.1.10. Primary Caregiver

Caregiving was often delegated to nonparental figures such as grandparents or older siblings, which may compromise the consistency and quality of care.

##### 3.2.1.11. Caregiving Quality and Quantity

Overburdened caregivers, high child‐to‐caregiver ratios, and limited individualized attention were prevalent, contributing to malnutrition and delayed healthcare‐seeking behavior.

Findings across these themes suggest strong interconnections; for example, the resource dilution observed in large families is compounded by limited caregiving quality. Such synergies between risk factors likely amplify children’s vulnerability to Noma.

## 4. Discussion

In this discussion, we examine each of the eleven family pathology themes, integrating evidence from both Noma‐specific research and broader child health literature. Each subsection explores the relevance of these factors for children diagnosed with Noma.

### 4.1. Stage of Child Psychological Development

Noma primarily affects children aged 2–6 years, a developmental stage in which dependence on caregivers for nutrition, hygiene, and emotional security is high [1, 7, 13]. At this stage, neglect or inconsistent caregiving can quickly deteriorate a child’s health. Early malnutrition and infections not only compromise physical growth but also impair cognitive development, as demonstrated by studies linking poverty to reduced brain surface area [12, 13].

The trauma of Noma, including pain, disfigurement, and stigma, further disrupts development. Approximately one‐third of survivors experience mental health conditions such as depression or posttraumatic stress disorder. These psychological effects are often worsened by isolation and community rejection, which undermine the child’s emotional security. Interventions aimed at improving family dynamics can reduce emotional pathology in vulnerable children [10].

Family‐level care deficits are common. In some cases, maternal absence and caregiving by overburdened grandmothers delay both emotional and cognitive development [14, 16]. Delayed recognition of symptoms can heighten disease severity, especially when stigma causes families to withdraw support [1, 10]. Responsive and emotionally attuned caregiving is a well‐established protective factor for children facing adversity [15]. Stein et al. (2014) further emphasize that maternal mental health plays a critical role in child development, particularly during the early years when brain plasticity and social bonding are most sensitive [19].

Noma’s onset in early childhood, when brain and social development are most active, means that family dysfunction can have lasting consequences. To mitigate this impact, interventions must prioritize caregiver education, early detection, and the integration of psychosocial support within community and healthcare systems [13, 20, 21].

### 4.2. Family Setting (Structure and Environment)

The family setting refers to the household structure, such as nuclear, extended, monogamous, or polygamous, which influences caregiving dynamics [11]. Noma‐affected children often come from nonnuclear settings, such as polygamous or extended families [6]. Polygamous households introduce complexity in spousal and parental roles, which can lead to conflict and dysfunction if not well managed [22, 23].

In polygamous households, competition among co‐wives may dilute maternal attention. Rivalry often leads to inconsistent or unequal caregiving, where some children’s needs are prioritized while others are neglected. Younger mothers may leave infants in the care of grandmothers to compete for their husband’s favor [22, 23]. While grandmothers can offer essential support, they may rely on outdated caregiving practices and often lack up‐to‐date knowledge about nutrition, hygiene, and disease prevention [14]. Studies consistently show that polygamous family structures are associated with higher child mortality, partly due to competition over resources and uneven distribution of care [22, 24].

Extended families, widely prevalent in sub‐Saharan Africa, can enhance supervision but also risk neglect if caregiving responsibilities are poorly defined or distributed across multiple adults. Baratti‐Mayer et al. identified a “crowded family entourage” as a significant risk factor for Noma [7].

Noma tends to emerge in complex, overcrowded, or polygamous settings where caregiving is inconsistent or inadequate. Preventive interventions must be culturally sensitive and target all household members, especially key caregivers such as grandmothers [7, 14, 23].

### 4.3. Family Location (Living Area)

Noma predominantly affects children living in remote, rural regions of the “Noma belt” in West and Central Africa, where poverty, limited infrastructure, and isolation converge to create conditions conducive to disease [1]. Recent data from Nigeria underscore this reality: in a 2024 case series, over 96% of children with Noma were from rural communities [25]. Rural residence is associated with limited access to healthcare, poor sanitation, and food insecurity, all of which heighten vulnerability to Noma [7, 25].

Families in rural settings are often excluded from vaccination campaigns and health education programs due to physical and infrastructural barriers [26]. Low vaccination rates and reliance on traditional healers further increase disease risk [7]. In many cases, beliefs in supernatural causes delay timely medical intervention and reinforce harmful stigma [10].

Although children in urban areas also live in poverty, they generally have better access to healthcare and immunizations than their rural counterparts. Studies have consistently shown that rural children experience significantly lower vaccination coverage, primarily due to greater distances from health services [26]. Broader research further highlights that rural populations face higher child mortality and lower life expectancy, largely due to limited access to basic services [27]. These disparities are particularly relevant in the context of Noma, as historical improvements in sanitation, nutrition, and healthcare infrastructure in high‐income countries were instrumental in eradicating the disease during the 20th century [1, 28].

Geographic isolation and rural poverty thus create a “perfect storm” for the emergence and persistence of Noma. Public health strategies must prioritize rural outreach, investing in infrastructure, improving access to care, and delivering culturally adapted health education tailored to the realities of rural families [25–27].

### 4.4. Family Income (Socioeconomic Status)

Poverty is central to the pathogenesis of Noma. It underlies key risk factors such as malnutrition, inadequate living conditions, and poor health literacy [1, 3]. Families experiencing chronic economic hardship often face food insecurity, resulting in undernutrition, a major risk factor for Noma. Dietary diversity plays a protective role: Regions with more varied food intake show lower Noma prevalence [7].

Economic deprivation also delays healthcare access. Families living in poverty often lack resources for transportation or medical expenses, resulting in advanced disease at the time of presentation [25]. Beyond physical deprivation, poverty is linked to reduced parental education and limited cognitive stimulation in children, exacerbating developmental delays [12, 29].

The family stress model suggests that economic hardship induces psychological stress in parents, which diminishes caregiving quality and responsiveness [9]. Long working hours in survival‐based labor reduce time for attentive childcare, often leaving children in the care of siblings who lack the maturity and skills required [30].

Sustainable Noma prevention must address the underlying socioeconomic conditions by combining public health interventions with broader social protection strategies aimed at reducing extreme poverty [5, 25].

### 4.5. Family Size (Number of Children and Household Size)

Large family size is a prominent feature among households affected by Noma [4]. Baratti‐Mayer et al. found that the risk of Noma increased with each additional child in the household. High maternal parity contributes to resource dilution, limited food availability, reduced maternal attention, and early weaning, all of which predispose children to malnutrition [7].

Studies have shown that larger families are associated with higher undernutrition rates due to constrained household resources [31, 32]. In crowded households, the risk of infection also increases, as close proximity among family members facilitates the spread of contagious diseases [33].

When many young children are present in the home, parents often struggle to monitor hygiene, nutrition, and signs of illness adequately. As a coping strategy, caregiving is sometimes delegated to older siblings, who may lack the maturity or knowledge to respond appropriately to health risks. From a developmental perspective, larger families reduce the amount of individualized attention each child receives [14, 31, 34]. Parental time per child decreases significantly as family size increases, particularly in low‐income households [35].

High maternal parity is also frequently associated with younger maternal age and low educational attainment, both of which exacerbate developmental and health risks. In many poor, rural areas, high birth rates are driven by limited access to family planning services and cultural norms that favor large families [7, 32, 36].

Reducing the burden of Noma requires culturally sensitive interventions that address both family size and caregiving capacity. Strategies should include improved access to contraception, education on birth spacing, and targeted support for large households, particularly through nutrition programs and trained community health workers [7, 25, 32, 36].

### 4.6. Level of Education of the Parents

Parental education, especially maternal education, is a fundamental determinant of child health and developmental outcomes. Higher levels of maternal education are consistently associated with greater uptake of maternal and child health services, such as prenatal care and immunizations, both of which are crucial in preventing diseases like Noma [36–38]. Educated parents are also more likely to seek timely medical care and adopt hygienic practices, thereby reducing the risk of infectious disease transmission [37, 38].

In Noma‐endemic settings, caregiving is sometimes delegated to extended family members, such as grandmothers. However, limited formal education among these caregivers may hinder the implementation of optimal health and nutrition practices. Education further shapes health beliefs and treatment decisions: Parents with formal education are less likely to attribute illnesses to supernatural causes and more inclined to pursue biomedical healthcare [10, 14, 37].

Parental education is also positively associated with children’s psychological development and coping capacity. For example, children of educated caregivers are more likely to demonstrate emotional resilience and stronger adaptive responses to stress [12, 15].

In addition, oral health practices, such as regular mouth cleaning, are more commonly observed in households with higher levels of parental education, reducing the risk of oral infections that may lead to Noma [37, 39]. Paternal education plays a vital supporting role; fathers with higher educational attainment are more likely to participate in family health decisions and ensure consistent care [20, 38].

Improving parental access to education, through both formal schooling and community‐based learning, is essential to breaking intergenerational cycles of poverty and disease and thereby reducing the risk of Noma [37, 38].

### 4.7. Parental Viability (Presence and Health of Parents)

Parental viability refers to the presence, physical health, and caregiving capacity of parents, all essential for promoting healthy child development [13, 19]. In Noma‐endemic regions, the absence or illness of a primary caregiver can severely undermine a child’s nutritional status, immune resilience, and access to healthcare [10, 13]. Children who experience the loss of a parent or live with chronically ill or emotionally unavailable caregivers are often at greater risk of malnutrition and preventable infections [13, 19].

Paternal absence, whether due to labor migration, family separation, or polygamous structures, may further strain caregiving resources. Research shows that children in such contexts are more likely to exhibit psychological distress, including anxiety and emotional insecurity [15, 40, 41]. Disruptions in caregiving continuity, particularly during critical developmental periods, may increase vulnerability to diseases of neglect such as Noma.

Nevertheless, studies underscore that consistent and nurturing caregiving, even by nonparental figures, can foster resilience and buffer children from adverse outcomes [42]. In this context, community‐based programs that support substitute caregivers and single parents through nutritional interventions, psychosocial support, and improved healthcare access are essential [11, 13, 19].

### 4.8. Marriage Issues or Conflicts

Marital discord, including interspousal conflict in both monogamous and polygamous marriages, is a well‐established contributor to family dysfunction and child neglect in contexts where Noma remains endemic. In polygynous households, rivalry among co‐wives often leads to fragmented caregiving, inconsistent emotional support, and destabilized family structures [8, 23, 43]. Empirical studies demonstrate that polygamy is linked to elevated levels of maternal depression, increased family conflict, and poorer physical and psychological outcomes in children [23, 43]. Polygamous marriages significantly increase the risk of depression, anxiety, and emotional insecurity among both mothers and children [43].

However, even in monogamous families, persistent marital conflict undermines caregiving quality. Children exposed to ongoing parental disputes are more likely to experience emotional dysregulation, behavioral problems, and increased vulnerability to infectious diseases, including Noma, via stress‐induced immune suppression [44, 45]. Laboratory‐based studies confirm that hostile marital interactions activate proinflammatory pathways and delay immune recovery, leading to slower wound healing and compromised immunity [45, 46].

Domestic violence further compromises maternal mental health and caregiving capacity. Stein et al. highlighted that perinatal mental disorders, particularly maternal depression, are strongly associated with reduced responsiveness and nurturing, affecting child development across multiple domains [19].

Marital conflict, whether in monogamous or polygynous households, undermines maternal mental health and caregiving, increasing children’s vulnerability to poor developmental and health outcomes, including Noma. Co‐wife rivalry, domestic violence, and persistent spousal discord contribute to emotional neglect and stress‐induced immune suppression. Addressing these psychosocial factors is essential for effective prevention strategies [19, 43–45].

### 4.9. Family Separation (Divorce, Abandonment, and Migration)

Family separation, whether due to divorce, abandonment, or labor migration, disrupts caregiving continuity and significantly compromises child health and development. Single‐parent households, particularly those led by women, are disproportionately affected by poverty, food insecurity, and limited access to healthcare and emotional support [41, 43, 47–49]. These conditions increase the risk of neglect, undernutrition, and poor hygiene, all key contributors to Noma.

Although joint custody arrangements may mitigate some negative outcomes by preserving children’s access to both parents’ resources, high‐conflict separations exacerbate psychological stress and undermine developmental stability [48, 50]. Parental labor migration, especially paternal absence, is common in many Noma‐endemic regions and deprives children of daily supervision and support. Multiple studies have shown that left‐behind children experience higher levels of depression, anxiety, and behavioral problems [41, 42, 49].

Inconsistent caregiving, such as rotating placement with extended family members, further destabilizes children’s emotional security. Chronic stress caused by separation weakens immune responses, increases susceptibility to infection, and heightens the risk of neglect and malnutrition. Historical accounts describe Noma outbreaks during periods of war, famine, and displacement, conditions often marked by family fragmentation [1, 16, 28, 34]. More recent evidence indicates that children in migrant‐sending households face a higher risk of suicidal ideation and mental health disorders [41].

To mitigate these risks, support systems for separated and single‐parent families are essential. These should include nutritional programs, psychosocial services, and community‐based child monitoring. Promoting family stability and providing targeted aid to vulnerable households can help break the chain of adversity that leads to Noma and related child health crises [10, 21].

### 4.10. Who Cares for the Child (Primary Caregiver)

The identity and competence of a child’s primary caregiver are pivotal for health and developmental outcomes [16, 20]. In Noma‐endemic regions, caregiving is frequently assumed by grandmothers, older siblings, or other relatives when mothers are absent [1, 14, 16]. While grandmothers provide valuable cultural and experiential knowledge, their caregiving practices may rely on traditional methods that are not aligned with modern health guidelines, increasing the risks of malnutrition and poor hygiene [14].

Older siblings often take on caregiving responsibilities despite lacking the maturity or knowledge to ensure consistent care and health monitoring. The evolutionary concept of allomothering, where caregiving is shared among nonmaternal figures, highlights that human child survival has historically depended on broad networks of care beyond the biological mother [24].

Informal caregivers, including grandparents and older siblings, often assume primary caregiving responsibilities in Noma‐affected households. These caregivers may be overburdened, lack formal education, or have limited knowledge of nutrition and hygiene practices, which can compromise the quality and consistency of care [14]. Evidence indicates that such caregiving burden can lead to delayed recognition of early symptoms, suboptimal nutrition, and increased risk of infection, all of which may exacerbate disease severity in Noma survivors [15]. Furthermore, the psychosocial stress experienced by informal caregivers may indirectly affect children’s emotional well‐being and recovery [16]. Interventions targeting caregiver support, education, and resource provision are therefore essential for improving outcomes in Noma‐affected children [14–16].

Attachment theory emphasizes that consistent, sensitive caregiving fosters secure emotional and physical development. Disruptions in caregiving, such as frequent shifts between mother, grandmother, and sibling, can lead to insecure attachment, developmental delays, and increased vulnerability to infections such as Noma [13, 16]. Moreover, contextual stress negatively impacts maternal sensitivity, which is a key predictor of responsive caregiving, as demonstrated in recent meta‐analyses [34].

Importantly, intervention studies confirm that grandparent caregivers can benefit from structured support. Randomized controlled trials and preventive programs aimed at grandparents have shown promising results in improving caregiving skills and child health outcomes [51].

Ensuring that every child in Noma‐prone settings has a stable, knowledgeable, and emotionally responsive caregiver, regardless of the caregiver’s identity, is essential for preventing neglect and promoting healthy development [13, 52].

### 4.11. Quality and Amount of Time Spent Caring for the Child

Both the quality and quantity of caregiving time are critical to child health. Research shows that, while parents intend to prioritize childcare, economic pressures severely limit the time available for focused caregiving [30]. In Noma‐endemic regions, caregivers are often overwhelmed by subsistence labor, which reduces their capacity to provide attentive care. Even when children are physically nearby, a lack of engaged interaction and observation may delay recognition of early signs of illness [13, 53].

Evidence from low‐ and middle‐income countries indicates that responsive caregiving, characterized by verbal engagement, emotional sensitivity, and consistent attention, substantially improves children’s nutritional, emotional, and cognitive outcomes [20]. In contrast, children cared for by overburdened or underprepared caregivers (e.g., siblings or elderly relatives) face a higher risk of neglect, developmental delays, and infection [16, 20].

Socioeconomic hardship is strongly associated with reduced caregiver–child interaction that promotes development [9, 47]. Neurodevelopmental research reveals that children from low‐income families exhibit reduced cortical surface area in brain regions critical for language and executive function, differences largely mediated by parental education and interaction quality [12].

Fortunately, caregiver training programs have demonstrated the potential to reduce these risks. Randomized trials show that educating caregivers in health monitoring and psychosocial support enhances caregiver self‐efficacy and encourages earlier health‐seeking behavior [54]. Community‐based interventions, such as UNICEF’s “Care for Child Development” program, have successfully integrated responsive caregiving messages into existing health and nutrition services [52].

To lessen the burden of Noma, strategies must support caregivers not only with information but also with time‐saving infrastructure, such as clean water access, proximity to clinics, and cooperative childcare options. Effective prevention depends on ensuring that caregivers have both the time and knowledge to meet children’s developmental and health needs [26, 53, 55].

## 5. Limitations

This scoping review has several limitations. First, although the literature search was extensive, relevant studies may have been missed due to indexing limitations, variations in terminology, or the exclusion of non‐English publications. Second, while the core search strategy captured Noma‐specific studies, these were often limited in scope and predominantly focused on biomedical aspects, with relatively little detail on family‐level or psychosocial determinants. To address these gaps, broader child health and family research was deliberately integrated. Although this approach enriched the thematic analysis, it also introduces interpretive assumptions, as such studies were not always conducted in Noma‐affected populations. Third, the heterogeneity of study designs and contexts limited the ability to perform a quantitative synthesis or directly compare findings across studies. Instead, a narrative synthesis was employed, which, while appropriate for scoping objectives, remains subject to interpretive bias. Finally, reliance on published, peer‐reviewed literature excludes insights from gray literature or community‐level reports, which may contain valuable but less systematically documented perspectives on family and caregiving dynamics.

## 6. Conclusion

Although Noma is clinically a gangrenous disease, its roots lie deeply in family and societal dysfunction. Affected children are typically very young, developmentally vulnerable, and raised in impoverished rural households with limited caregiving resources. Care is often delegated to grandmothers or older siblings who may lack the knowledge, skills, or time to provide attentive care. Marital discord, caregiver absence, and family separation further weaken the child’s protective environment.

Importantly, Noma is a preventable condition. Strengthening families through caregiver education on nutrition, hygiene, and early signs of Noma, along with promoting birth spacing and implementing economic support interventions, is crucial. Collaborating with traditional and religious leaders may also help to challenge and reduce harmful cultural practices.

Improving healthcare access through community health workers and integrating Noma screening into primary care services, combined with broader efforts to improve food security, are essential strategies. Psychosocial support, delivered through parenting programs and family‐centered rehabilitation, can facilitate reintegration and reduce the stigma experienced by survivors and their families.

Ultimately, preventing Noma requires holistic strategies that address both poverty and family dysfunction. Investing in resilient, empowered families ensures that children grow up healthy. Where families thrive, Noma disappears.

## Funding

This research received no specific grant from any funding agency in the public, commercial, or not‐for‐profit sectors.

## Conflicts of Interest

The authors declare no conflicts of interest.

## Supporting Information

Additional supporting information can be found online in the Supporting Information section.

## Supporting information


**Supporting Information** Only one supporting file accompanies this article: Supporting Information 1: Detailed Search Strategy. Databases searched (PubMed and MEDLINE; from inception through May 2024), date last searched (May 2024); core Noma‐specific PubMed string and example broader child‐health queries; additional source identification via reference‐list screening and cross‐referencing; eligibility criteria (peer‐reviewed, PubMed‐indexed; English; specified study types); and selection process (dual‐review screening with consensus), as outlined in the Methods.

## Data Availability

All data supporting this review are available in the included published studies. No new datasets were generated or analyzed.
