# Supplementary material for: The Role of Family Pathology in Noma: A Scoping Review of Household‐Level Risk Factors in Sub‐Saharan Africa
Source: J Trop Med. 2026 May 27;2026:4767171. doi: 10.1155/jotm/4767171 (PMC13216661; doi:10.1155/jotm/4767171)
Supplement: Supplementary file 1 — Supporting Information Only one supporting file accompanies this article: Supporting Information 1: Detailed Search Strategy. Databases searched (PubMed and MEDLINE; from inception through May 2024), date last searched (May 2024); core Noma‐specific PubMed string and example broader child‐health queries; additional source identification via reference‐list screening and cross‐referencing; eligibility criteria (peer‐reviewed, PubMed‐indexed; English; specified study types); and selection process (dual‐review screening with consensus), as outlined in the Methods. [file JOTM-2026-4767171-s001.docx]

**Supplementary Material 1 – Detailed Search Strategy**

**Databases searched:**

PubMed and MEDLINE (from inception through May 2024).

**Date last searched:**

May 2024.

**Core Noma-specific search string (PubMed example):**

("Noma"[MeSH] OR "Noma" OR "cancrum oris") AND ("family" OR "socioeconomic" OR "risk factors" OR "parental" OR "caregiver" OR "poverty" OR "psychosocial")

**Supplementary search strings (broader child health determinants):**

These were used to capture evidence on family and caregiving contexts beyond Noma-specific studies. Examples include:

("family size" AND "malnutrition" AND "child")

("parental education" AND "child health")

("marital conflict" AND "child development")

("caregiving quality" AND "child nutrition")

("family separation" AND "child outcomes")

**Additional sources:**

- Manual screening of reference lists of included Noma studies.

- Cross-referencing of included articles to identify supplementary child health studies that address family-level determinants relevant to the review’s objectives.

**Eligibility criteria applied to retrieved results:**

- Peer-reviewed studies indexed in PubMed.

- Epidemiological, case-control, case series studies, or reviews addressing Noma risk factors.

- Studies (including reviews and meta-analyses) on child health and family environment (e.g., parental education, caregiving, family size, psychosocial stressors).

- English language only.

- Exclusion: grey literature, non-peer-reviewed reports, media articles.

**Selection process:**

- Two reviewers independently screened titles/abstracts.

- Full-text screening against eligibility criteria.

- Discrepancies resolved by consensus, with a third reviewer if needed.

**Notes:**

- Broader child health literature was deliberately included to fill thematic gaps left by Noma-specific research.

- This approach is consistent with the stated scope and objectives of the review, as clarified in the Methods section.
